# Human Processing of Behaviorally Relevant and Irrelevant Absence of Expected Rewards: A High-Resolution ERP Study

**DOI:** 10.1371/journal.pone.0016173

**Published:** 2011-01-27

**Authors:** Louis Nahum, Damien Gabriel, Armin Schnider

**Affiliations:** 1 Laboratory of Cognitive Neurorehabilitation, Department of Clinical Neurosciences and Dermatology, Medical School, University of Geneva, Geneva, Switzerland; 2 Division of Neurorehabilitation, Department of Clinical Neurosciences, University Hospital of Geneva, Geneva, Switzerland; University College London, United Kingdom

## Abstract

Acute lesions of the posterior medial orbitofrontal cortex (OFC) in humans may induce a state of reality confusion marked by confabulation, disorientation, and currently inappropriate actions. This clinical state is strongly associated with an inability to abandon previously valid anticipations, that is, extinction capacity. In healthy subjects, the filtering of memories according to their relation with ongoing reality is associated with activity in posterior medial OFC (area 13) and electrophysiologically expressed at 220–300 ms. These observations indicate that the human OFC also functions as a generic reality monitoring system. For this function, it is presumably more important for the OFC to evaluate the current behavioral appropriateness of anticipations rather than their hedonic value. In the present study, we put this hypothesis to the test. Participants performed a reversal learning task with intermittent absence of reward delivery. High-density evoked potential analysis showed that the omission of expected reward induced a specific electrocortical response in trials signaling the necessity to abandon the hitherto reward predicting choice, but not when omission of reward had no such connotation. This processing difference occurred at 200–300 ms. Source estimation using inverse solution analysis indicated that it emanated from the posterior medial OFC. We suggest that the human brain uses this signal from the OFC to keep thought and behavior in phase with reality.

## Introduction

Acute lesions of the posterior medial orbitofrontal cortex (OFC) or structures directly connected with it may induce a state of dramatic reality confusion in human subjects: The patients confabulate recent experiences that never took place, are disoriented, confusing the time, place, and their current role, and enact ideas (e.g., going to work) that do not apply to current reality [1], [2]. This state, variably called spontaneous confabulation [3], confabulation with action [4], or behaviorally spontaneous confabulation [2], emanates from an inability to suppress the interference of memories that do not relate to the present [3], [5], [6]. Lesions involve the posterior medial OFC (area 13 and ventromedial prefrontal cortex) or regions directly connected with it [1], [5], [6], [7], [8], [9]. In healthy subjects, the ability to filter out memories that do not relate to present reality (memory filtering) occurs at an early stage of memory evocation, at 220–300 ms [10]. It involves orbitofrontal area 13 and connected subcortical structures [11], [12] and is under dopaminergic modulation [13].

These observations show that the human OFC is critical for the ability to adapt thought and behavior to ongoing reality. Current theories on OFC functions offer no explanation for such a role. The OFC is seen as a hedonic and decision-making centre that optimizes behavior and choices on the basis of anticipated and obtained rewards [14], [15], [16], [17]. Indeed, single cell recordings in animals revealed neurons in the OFC whose discharge rate reflects the type [18], current value [16], [19], occurrence [20], [21] or omission [20], [22] of expected rewards [23]. A wealth of functional imaging studies in humans confirmed the OFC's role in the processing or rewards [24], [25], [26], [27], [28] and extended the notion of reward processing to abstract monetary reward [29], [30], [31]. Varying in details, these studies also showed an anatomical diversity of different aspects of reward processing in the OFC [30], [31], [32], [33], [34], [35]. In particular, the lateral OFC was shown to be involved in the coding of changes in reward contingencies during probabilistic reversal learning [35], [36]. Clinical studies, too, focused on the processing of rewards, mostly money, after OFC lesions [37], [38], [39] and did not consider an elementary faculty like reality filtering. This may be due to the fact that the state of reality confusion after acute OFC lesions is rare [2] and in most cases transitory: within a few months, most patients act again in agreement with reality and regain correct orientation in time and space [8].

A striking feature of this reality confusion is that patients continue to act according to ideas and plans that do not relate to the present. We have, therefore, speculated that their primary failure is an inability to adapt their thinking and behavior to the fact that their –currently inappropriate- anticipations fail to occur; the absence of expected outcomes fails to produce a signal indicating discordance between their ideas (thoughts) and reality [2]. The primate posterior medial OFC –the area damaged or disconnected in the patients– has a particularly high density of neurons that specifically fire when anticipated outcomes (rewards) fail to occur [20], [22]. In analogy to these observations in animals, we thus hypothesized that the reality confusion of our patients reflected absence of, or the inability to make use of, the orbitofrontal signal which would normally indicate the non-occurrence of anticipated outcomes, that is, the neural signal that normally underlies extinction [2]. We obtained critical support for this hypothesis in a clinical study: we found that disorientation and behaviorally spontaneous confabulation in patients with OFC lesions or amnesia were very strongly and specifically associated with a failure to abandon a previously correct choice in a reversal learning task once it was not followed by the expected outcome anymore [40]. In contrast, the ability to subsequently learn a new association was not predictive of orientation or behaviorally spontaneous confabulation.

These findings suggest that the OFC might be at least as important for processing the behavioral relevance as the hedonic value of outcomes. While activation of the posterior medial OFC in the processing of outcomes devoid of any tangible reward value [41] and early signaling of behaviorally relevant absence of outcomes at 200–300 ms in such a task [42] has been observed before, the processing of behavioral relevance and hedonic loss of the absence of anticipated rewards have never been directly compared. In the present study, we used high-resolution event-related potentials (ERP) to explore the electrocortical correlate of reward delivery and reward omission with two situations of reward omission: in one, the correctness of the previous choice was confirmed and behavior could continue as before; in the other, the hitherto correct choice had to be abandoned and a change of choice was required in the next trial. Thus, both outcomes lacked hedonic value but only one required abandonment of a previously correct behavior. We hypothesized that the absence of reward would induce an early electrocortical response (200–300 ms [10], [42]) only when it signaled a need to subsequently adapt behavior, while it would induce either no such signal, or at another point in time, when it had no behavioral relevance, that is, when the previously valid anticipation remained valid. Apart from traditional waveform analysis, we used advanced ERP topographic mapping techniques to estimate the generators of the electrocortical activity.

## Materials and Methods

### Participants

Eighteen right-handed healthy subjects (7 males, 11 females) aged 26±4.6 (mean ± SD) years gave written, informed consent to participate in the study. They were paid 20 Swiss francs per hour and could earn additional money based on their performance. The institutional Ethical Committee approved the study.

### Procedure and Task

**Figure 1:**
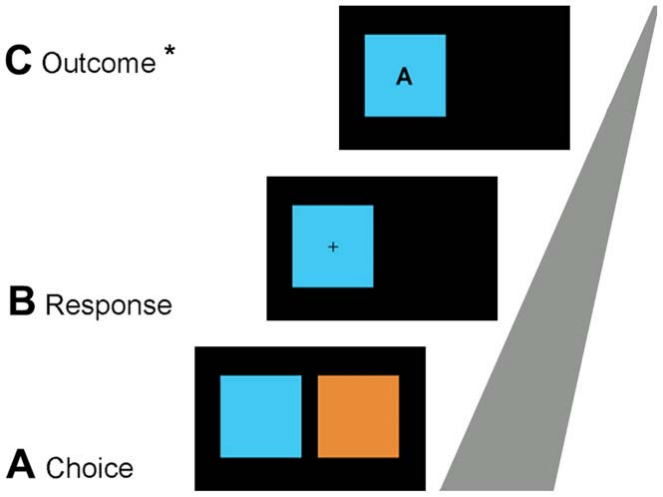
Design of the experiment. Trials had three steps: **A**, Two differently colored rectangles were presented and subjects had to predict by button press which one of the two rectangles hid a “gamble”. **B**, After the choice, only the chosen rectangle remained on the screen and a fixation cross appeared in its centre. **C**, 1500 ms later, one of three possible letters indicating the outcome appeared in the centre of the rectangle (outcome). The meaning of the letter was individually chosen and practiced before the start of the experiment (Table *1*). After 1000 ms, the screen turned black; 700 ms later, the next trial started. ERPs were time-locked to the appearance of the outcome stimulus.

Participants performed a simple probabilistic reversal learning task in which they had to predict behind which one of two colored rectangles a “gambling set”, that might provide reward or not, was hidden (Figure 1). Stimuli were presented on a black background on a 21-inch monitor with a resolution of 1024×768 pixels using e-prime (Psychology Software Tools, Pittsburgh, PA). Subjects were told that the “gamble” would normally remain behind the same rectangle but that it occasionally switched to the other rectangle. They should base their choice on the outcome of the last trial and refrain from guessing.

**Table 1:** Possible outcomes and their probability of occurrence.

Letter	Outcome type (Probability)		
	Reward(50%)	No-Reward(25%)	Extinction(25%)
**A**	**A**rgent*Money*	**A**bsent*Absent*	**A**rrêt*Stop*
**P**	**P**oints*Points*	**P**as*No*	**P**lus*No longer*
**S**	**S**ous*Coins*	**S**ans*Without*	**S**top*Stop*

For each subject, the three letters A, P, and S were used but their meaning was chosen individually for each subject and trained by repeated presentation. The table shows the letters' possible meanings in French (as used in the experiment) and their English translation.

Trials started by the appearance of the two colored rectangles (Figure 1A). After subjects had indicated their choice –the rectangle where they expected the gamble to play– by pressing a response key (right hand, index finger for the left-sided rectangle, middle finger for the right-sided rectangle), the non-chosen rectangle disappeared and a fixation cross appeared in the center of the chosen rectangle (Figure 1B). After 1500 ms, the outcome was presented in the form of a letter in the center of the rectangle (Figure 1C), whose significance had been practiced before the experiment. After 1000 ms, the screen turned black; 700 ms later, the next trial started with the appearance of the two colored rectangles.

Three letters (A, P, S), whose meaning varied between the subjects, indicated the outcomes: Two letters indicated that the subjects had actually chosen the correct rectangle, where the gamble was playing. One of these letters indicated that they received some money (5 cents, Reward trials). This was the most frequent trial type (50% of all trials). The second letter indicated that they had chosen the correct rectangle but that they obtained no reward (No-Reward trials, approx. 25% of all trials). The third letter indicated that there was no reward because the gamble was not playing behind the chosen rectangle anymore (approx. 25% of all trials, after 2 to 4 consecutive correct responses). As these trials signaled the need to abandon the chosen rectangle on the next trial, we called them Extinction trials, similar to our previous studies [40], [41], [42], [43]. These trials constituted the first phase of the reversal (switch) to the alternate rectangle, which was completed by the learning of the new stimulus-association on the next trial when the alternate rectangle was again visible.

Incorrect responses put the counter of a trial sequence back to zero; subjects again had to make 2 to 4 correct choices before an Extinction trial. Incorrect responses were not analyzed because of their scarcity (see Results). The main task consisted of three experimental blocks of 140 trials each.

The meaning of the letters signaling the three possible outcomes (A, P, S) varied and was counterbalanced between participants. Subjects familiarized with the letters' meaning referring to their session in 40-trial practice blocks of the “gamble” task. Rather than presenting only the letters, as in the main task, outcomes in the practice block were signaled by the highlighted letters completed to whole words (Table 1). Training was repeated until participants had less than 3 unforced errors and correctly reported the meaning of each letter.

### Analysis of behavioral data

The following behavioral data were obtained: Reaction times (time to choose one rectangle after appearance of the two colored rectangles; Figure 1A) and proportion of errors after the 3 trial types. In addition, participants indicated at the end of the experiment on a visual analogue scale (VAS, Likert scale from 1 to 10) how much they had liked the 3 outcome types (pleasantness score) and how often they had expected them to occur (degree of anticipation). These measures were compared using repeated measures ANOVAs. Post-hoc tests of simple effects were Bonferroni corrected.

### EEG acquisition and preprocessing

The electroencephalogram (EEG) was recorded continuously using the Active-Two Biosemi EEG system (Biosemi V.O.F Amsterdam, Netherlands) with 128 channels covering the entire scalp. Signals were sampled at 512 Hz in a bandwidth filter of 0.1–104 Hz. All analyses were conducted using Cartool Software (http://brainmapping.unige.ch/Cartool.htm). Epochs of EEG from 200 ms before to 800 ms after the onset of the outcome stimulus were averaged for each subject and each condition. In addition to a ±100 µV rejection artefacts criterion, EEG epochs containing eye blinks and movements or other sources of transient noise were excluded during the averaging procedure. Artefact electrodes were interpolated using a spherical spline interpolation [44]. Baseline correction was applied to the 200 ms prestimulus period. Before group averaging, individual data were recalculated against the average reference and bandpass filtered to 1–30 Hz. The number of evoked potentials (ERPs) entering the analysis was matched across conditions in each participant (mean ± SD per condition, 54±7; min. 40, max. 60).

### Waveform analysis

In order to allow comparison of our results with earlier studies, we first examined amplitude differences of ERP traces at nine electrode positions described in previous studies on outcome processing and covering anterior, central, and posterior regions of both hemispheres (corresponding to AF8, AF7, AFz, PO7, Pz, PO8, Oz, FCz and Cz of the International 10–20 System). To estimate periods of amplitude difference, we performed point-wise paired t-tests over 800 ms for every 2 ms interval following stimulus onset. Only differences extending over at least 20 ms at p<.05 (Bonferroni corrected by the number of electrodes –1) were retained and will be illustrated in the results section.

### Topographic analysis

Amplitude variations of ERP traces do not allow distinguishing between activation of different networks (with different potential fields) or modulation of similar networks [45]. We therefore applied a reference-free spatiotemporal analysis approach that searches for topographical differences of the global scalp potential maps between conditions across time [46], [47], [48]. Different map configurations indicate different intracranial generator distributions [49]. The approach is based on a modified spatial k-means clustering analysis [50] that determines the most dominant map topographies and the periods during which they are present in the data. This approach is based on the observation that scalp topographies do not change randomly, but rather remain for a period of time in a certain configuration and then rapidly switch to a new stable configuration [51], [52]. The periods of stability have been called “functional microstates” [51], [52], [53] and are thought to reflect the different information processing steps.

The cluster analysis was applied to the group-averaged ERPs of the three outcome types (Reward trials, No-Reward trials, Extinction trials). We further applied the constraint that a given scalp topography must be observed for at least 20 ms in the group-averaged data. Additionally, statistical smoothing was used to eliminate temporally isolated scalp topographies with low strength [50]. This topographic analysis method is independent of the reference electrode and is insensitive to amplitude modulation of the same scalp configuration across conditions, because topographies of normalized maps are compared [51]. The optimal number of maps explaining the averaged data sets was determined with the cross validation [50] and the Krzanowski-Lai criterion [54].

In a second step, the appearance of maps identified in the group-averaged data was statistically verified in the ERPs of the individual subjects. To do this, each map was compared with the moment-by-moment scalp topography of the individual subjects' ERPs from each condition by strength-independent spatial correlation [53], [55], [56]). That is, for each time point of the individual subjects' ERPs, the scalp topography was compared to all maps and was labeled according to the one with which it best correlated. It is important to note that this labeling procedure is not exclusive, such that a given period of the ERP for a given subject and stimulus condition is often labeled with multiple template maps. Nonetheless, the results of the labeling reveal whether a given ERP is more often described by one map rather than another. Fitting thus allowed us to determine for what period of time a given topography was observed in a given condition across subjects. The Global Explained Variance (GEV) is the sum of the explain variance weighted by the Global Field Power (GFP, root mean square across the average-referenced electrode values at a given instant in time [53], [56]). The GFP represents the strength of the maps. The GEV describes how well a map configuration explains the individually obtained patterns of activity [45], [53]. The GEV and duration of maps were then subjected to repeated measures ANOVA using outcome type (Reward trials, No-Reward trials, Extinction trials) and map as within-subject factors. P-values of post-hoc single comparisons were Bonferroni corrected.

### Source localization

In order to estimate the brain regions accounting for the different electrocortical map configurations, source localization was applied using a distributed linear inverse solution based on a Local Auto-Regressive Average (LAURA) model comprising a solution space of 3005 nodes [57]. Current distribution was calculated within the grey matter of the average brain provided by the Montreal Neurological Institute (MNI). Similar to statistical parametric mapping (SPM) used in fMRI studies, we computed the contrasts of local electrical current densities between the three outcome types with time-point wise paired t-tests in the periods in which the map configurations significantly differed, that is, 200–300 ms and 485–635 ms. P values were Bonferroni corrected by the number of electrodes, so that only nodes with p<.0004 for at least 20 ms were retained [45].

## Results

### Subjective ratings

Subjects consistently preferred the letter signaling reward (VAS, 6.7±2.6, mean ± SD) over the two letters indicating absence of reward (No-Reward, VAS, 3.4±1.9; Extinction, VAS, 4±2.7; *F*(2,34)  = 14.278, p = .00003), the latter two obtaining similar pleasantness scores. They reported having differently anticipated the outcome types (ANOVA, *F*(2,34)  = 5.33; p = .009). Reward trials (VAS, 7±1.4) were more anticipated than No-Reward trials (5.3±2) (p = .007) and Extinction trials (5.5±2.5) (p = .02), whereas No-Reward and Extinction trials were equally anticipated (p = .65).

### Task performance

The task proved very easy: subjects made only 1.8±1.2% (mean ± SD) unforced errors. The outcome type of the previous trial influenced accuracy (ANOVA, F(2,34) = 42; p<.001) and reaction times (F(2,34) = 6.6; p = .004). Participants made more errors after Extinction trials (3.5±2.2%) than after Reward trials (0.02±0.07%; p<.001) and No-Reward trials (0.14±0.3%; p<.001). Trials following No-Reward trials and Reward trials did not differ (p = 0.78). Reaction times (response latencies) were longer in trials following an Extinction trial (570±102 ms) than after Reward trials (542±100 ms; p = 0.03) and tended to be longer than after No-Reward trials (555±102 ms, p = 0.1). Trials following No-Reward and Reward trials did not differ (p = 0.37).

### Waveform analysis

**Figure 2:**
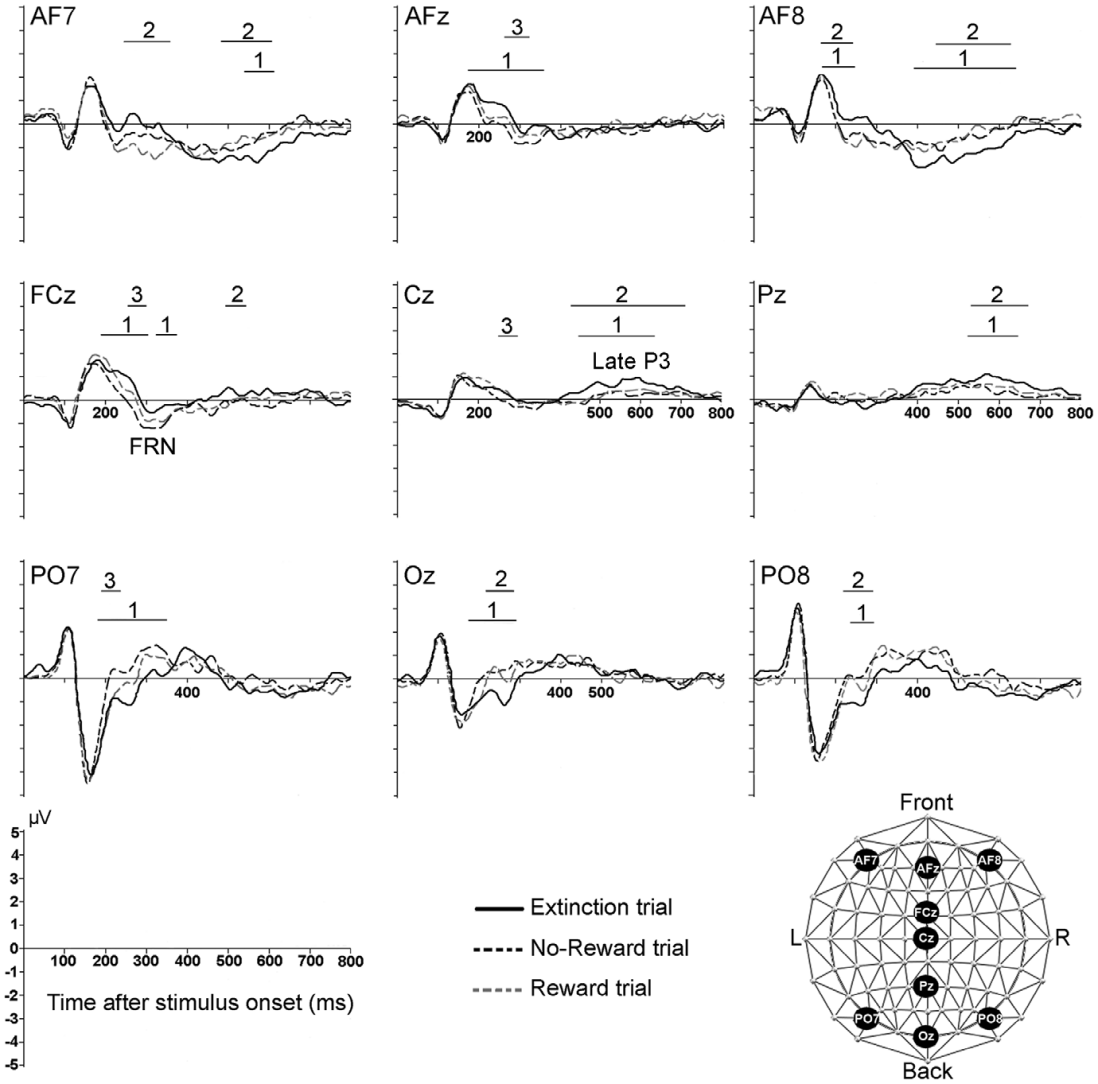
Evoked potential curves in response to the three outcome types. Periods displaying significant amplitude differences between two outcome types over at least 20 ms are indicated with bars. Numbers above the bars indicate significant differences between: 1, Extinction vs. No-Reward trials; 2, Extinction vs. Reward trials; 3, Reward vs. No-Reward trials. The position of the corresponding electrodes is shown at the bottom right. FRN, feedback-related-negativity.

Analysis of waveforms indicated two main periods of significant amplitude differences after stimulus onset: approximately 200–300 ms and 450–650 ms (Figure *2*). Extinction trials induced a more positive frontal (AF7, AFz, AF8, FCz) and negative posterior (PO7, Oz, PO8) response than No-Reward and Reward trials between 200–300 ms. Towards the end of this period, around 300 ms, No-Reward trials elicited a typical feedback-related negativity (Figure 2) characterized by a more negative deflection at frontal electrodes (AFz, FCz) and a more positive deflection at lateral posterior electrode PO7 than Reward trials and Extinction trials (200 and 300 ms).

Between 450–650 ms, Extinction trials elicited a more negative lateral frontal (AF7, AF8) and more positive central responses (Cz, Pz) than the two other trial types, corresponding to a late P3 (Figure 2).

### Topographic analysis

**Figure 3:**
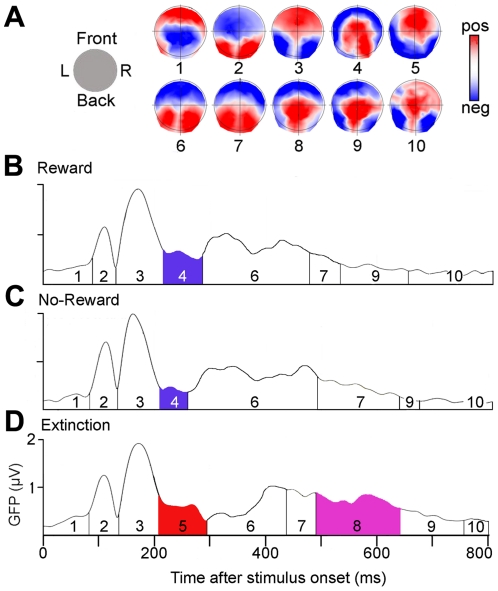
Electrocortical map configurations in response to the different outcomes. **A**, Cortical maps obtained by segmenting the grand-mean of the ERPs between 0 and 800 ms. **B–D**, sequence of the maps between 0 ms and 800 ms after outcome presentation for each condition and map strength expressed as the Global Field Power (GFP). **B**, Reward trials; **C**, No-Reward trials; **D**, Extinction trials. Maps with significantly different Global Explained Variance (GEV, a measure of how well a map explains individual data) between the conditions are shown with colored areas under the curves.

Spatio-temporal segmentation yielded 10 distinct potential map configurations over 800 ms (Figure *3A*). Figure *3B–D* shows the sequence of the dominant maps at any moment and the relative strength of the maps (GFP) in response to the three outcome types. The earliest and most striking difference appeared at 200–300 ms, when Extinction trials evoked a configuration (map 5 in Figure *3D*) having opposite anterior-posterior polarity to the one evoked by Reward and No-Reward trials (map 4 in Figure *3B–C*). Statistical analysis confirmed an interaction of map X outcome type regarding the presence of the two maps (Global Explained Variance, GEV; *F*(2,34)  = 7.57, p<.001). Post-hoc tests confirmed the stronger presence of map 5 in Extinction trials. Between 300 and 485 ms, all three outcome types evoked the same map configurations, although one configuration (map 6 in Figure *3*) was significantly longer present in Reward (Figure *3B*) and No-Reward (Figure *3C*) than Extinction trials (Figure *3D*) (*F*(2,34)  = 13.33, p<.001).

Map configuration again significantly differed between 485 and 635 ms, when Extinction trials evoked a different configuration (map 8 in Figure *3D*) than Reward and No-Reward trials (maps 7 and 9). This difference was confirmed by a significant interaction of map (maps 7, 8, 9, 10) X outcome type (GEV, *F*(6,102)  = 6.7, p = .00001), which was due to a stronger presence of map 8 in Extinction trials.

Differences between Reward and No-Reward trials were discrete, the only significant difference being a longer duration of an early map (map 4) between 200 and 300 ms in response to Reward trials (*F*(1,17)  = 6.1, p = .02) and of map 7 in response to No-Reward trials than the two other conditions (*F*(6,102)  = 7.1, p = .001).

### Source localization

**Figure 4:**
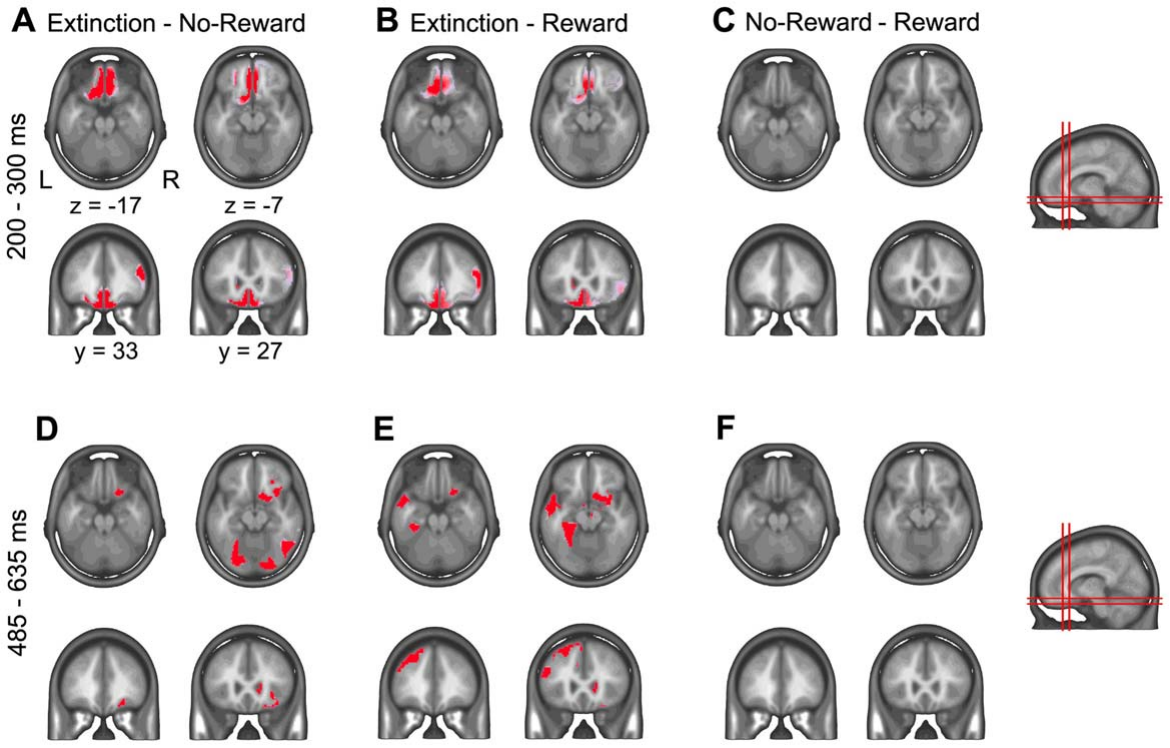
Inverse solution. Areas with significantly different current densities as determined from group-averaged source estimations during the time periods 200–300 ms (**A–C**) and 485-635 ms (**D–F**) for the following contrasts: **A,D**, Extinction – No-Reward; **B,E**, Extinction – Reward; **C,F**, Reward – No-Reward. Red areas depict solution points with statistically significant differences (p<.0004 for at least 20 ms) are depicted in red on axial and coronal slices of the brain template of the Montreal Neurological Institute. Coordinates x and z are given in Talairach space.

Figure *4A,B* shows that, between 200 and 300 ms, Extinction trials induced significantly stronger activation than both Reward and No-Reward trials in the posterior medial OFC, extending to area 13, 10, 11, and 14. Between 485–635 ms, Extinction trials induced stronger activation of the right posterior lateral OFC (area 47/12). In addition, in this late period, Extinction trials induced extended, left-sided inferomedial temporo-occipital activity, including the medial temporal lobe (Figure *4D,E*). No area was more active in Reward or No-Reward trials than Extinction trials. Areas activated by Reward and No-Reward trials did not significantly differ in either period (Figure *4C,F*).

## Discussion

The present study indicates that the behavioral relevance of an outcome may be a stronger driver of early human cerebral activity (and OFC activity in particular) than hedonic value. Absence of reward elicited strikingly different electrocortical responses when it signaled that the previously reward-predicting choice was no longer valid (Extinction trials) than when it simply indicated non-delivery of reward despite correct choice (No-Reward trials) or when reward was delivered (Reward trials). Specifically, Extinction trials evoked distinct electrocortical responses already at an early stage of processing between 200–300 ms, which were evident in the waveform analysis (Figure *2*) and induced a significantly different overall electrocortical map configuration (Figure *3*). Source estimation indicated that this difference emanated from stronger activity of the posterior medial OFC in Extinction trials than the other trial types (Figure 4).

The specific response to Extinction trials is in remarkable agreement with an earlier study, in which subjects had to anticipate “behind” which one of two colored rectangles an “object” was hidden. However, in contrast to previous studies on outcome processing and the present study, no reward was involved: subjects received no comment, no score, and no other form of reward at the end of trials [42]. Despite absence of any notion of reward, trials requiring a switch to the other rectangle in the next trial evoked a specific electrocortical response with a similar configuration as observed in the present study: there was a strong positive potential over frontal electrodes and a specific map configuration (with frontal positivity) at 200–300 ms. No such potential was present when an unexpected but irrelevant change of outcome occurred, namely, presentation of another object. The present study shows that, when a gamble is about obtaining reward or not, behavioral relevance of the absence of an outcome is a stronger driver of electrocortical activity than the sole absence of the expected reward.

The markedly different response specific to Extinction trials cannot be due to differences in stimulus properties (such as, the use of “$$” symbols or numbers), as all outcomes were signaled by single letters whose significance was initially learned and which varied between participants.

No-Reward and Reward trials induced only discretely different responses, although these outcomes differed both with regards to probability of occurrence and hedonic value: No-Reward trials induced a more prominent frontal negativity around 300 ms characteristic of the feedback-related negativity (FRN, Figure 2), which is thought to reflect an erroneous or disadvantageous choice [58], [59], [60], [61]. This processing difference did not induce a significantly different overall electrocortical map configuration (Figure 3). Of note, in contrast to No-Reward trials, Extinction trials did not induce a FRN. This observation is in agreement with a recent study in which outcomes that preceded behavioral adjustment in a probabilistic learning task did not induce a FRN [62]. The finding underscores the idea that, in a situation in which the non-occurrence of reward may or may not have behavioral relevance, as in our task, the electrocortical response to the behaviorally relevant absence of an outcome overrides the effect of the simple processing of a disadvantageous outcome. Hence, the strong frontal positivity induced by the processing of the behavioral relevance inherent in Extinction trials may have prevented the appearance of a FRN in response to these trials.

There was a second period, around 450–700 ms, when Extinction trials induced a stronger late P3 component compared to other trial types and a specific electrocortical map configuration (Fig. 2,3D). This potential might reflect the determination to adapt behavior in the subsequent trial, as a late P3 was also observed in endogenously generated shifts of the perceptual rule during the Wisconsin Card Sorting test (WCST) [63]. Source estimation indicated that the trace and map differences reflected stronger activity of right lateral OFC (area 47/12) and the left medial temporal lobe (MTL, Fig. 4D,E); again, there was no significant difference between No-reward and Reward trials. This activity might be explained by the fact that our task was a reversal task, in which Extinction trials not only indicated that the current behavioral choice had to be abandoned (as in a pure extinction task), but also that an alternative behavior was required in the next trial. In primates, lateral orbitofrontal lesions induced a specific deficit of object alternation [64]. Similarly, human functional imaging showed activity of the lateral right OFC in reversal learning [65], [66]. A recent lesion study in monkeys performing an analog of the WCST supported these interpretations: lesions of the OFC impaired rapid reward-based updating of representations of rule value –corresponding to the rapid processing of the behaviorally relevant absence of an expected outcome in the present study–, while ventrolateral prefrontal lesions impaired implementation of previously acquired abstract rules –the behavioral switch in our study [67].

The MTL activity at 450–700 ms might reflect encoding of the last relevant event (the rectangle not followed by the anticipated reward) or evocation of the memory of the alternate, currently invisible, stimulus. This interpretation is compatible with an earlier H_2_ [15]O PET study on reversal learning which showed stronger MTL activation when the outcome of trials was relevant for subsequent behavior than when subjects were asked to guess and the outcome of trials was irrelevant for subsequent choices [41].

The localization of brain activity in the present study –OFC and MTL– was based on source estimation using inverse solutions of high-resolution EEG. This technique is capable of localizing epileptic discharges emanating from the medial temporal lobe [68] and correctly localized MTL activity in healthy subjects performing a memory task [69], as confirmed by depth electrode recordings in epileptic patients performing the same task [70]. There is no theoretical reason to consider the OFC a less amenable region to this localization technique than the MTL, but formal proof is lacking. Nonetheless, there is strong evidence that this localization is correct: healthy subjects performing a similar task had strong activation of the posterior medial OFC [41]. This result was reliable as it was obtained with H_2_ [15]O PET which has no artifacts in this area, in contrast to fMRI, which is typically heavily distorted by susceptibility artifacts induced by the adjacent sinuses (normalization procedures may hide, but cannot compensate for these artifacts) [71], [72]. Most importantly, patients with lesions of the medial OFC have difficulty in abandoning a previously correct choice in reversal learning [39], a failure that is strongly associated with disorientation and behaviorally spontaneous confabulation in the acute phase [40]. Thus, the OFC localization of the critical signal in the present task was not really much of an issue; the study rather explored, in response to what type of outcome stimulus (hence an event-related method) and when (hence a rapid method) a specific brain response to outcomes would be observed. The fact that the inverse solution technique used here localized the main electrophysiological finding (specifically stronger response at 200–300 ms in Extinction trials) to the posterior medial OFC is, therefore, highly comforting and indirectly supports the localization potential of the method.

We used the term “Extinction trials” (rather than “switch” [73] or “reversal trials” [74]) in this paper because only the cued stimulus that no longer predicted reward was visible when the outcome was presented, but not the alternate stimulus (the other colored rectangle). Thus, these trials stressed the extinction phase of reversal learning (abandonment of the hitherto valid cue), while they did not show the stimulus with which the reward association had to be established in the next trial. Disorientation and behaviorally spontaneous confabulation are associated with failure in this first phase of reversal [40]. The term extinction was used in a generic sense, defined as the situation in which “one learns that certain expectations no longer apply” [75], as in our experiment. Pavlov had introduced the term to describe the weakening of a conditioned reflex when a conditioned stimulus was not followed by reinforcement [76]. This type of extinction has a known neural substrate in animals: A specific deficit of extinction was observed in monkeys with lesions of the posterior medial OFC, but not other parts of the frontal lobes [64]. Single cell recordings showed that this area contains a particularly high density of neurons that exclusively increase their discharge rate when anticipated reinforcements (rewards) fail to be delivered, that is, in trials whose repetition would induce extinction [20], [22]. Our hypothesis is that the brain uses this very signal, which is evoked when an anticipated outcome (reward) fails to occur, to keep thought and behavior in phase with reality [40], [77]. We suggest that this capacity relies on singular events, similar to the Extinction trials of our task, and does not require the repeated absence of reinforcement necessary for the extinction of a conditioned reflex. In anatomo-pharmacological terms, we suspect that the well-known orbitofrontal-subcortical reward circuitry [23], [78], which has also been shown to participate in reality filtering [12], [13], assumes among many other functions also the one of signaling when an upcoming thought does not relate to ongoing reality. The present study supports the idea that the human OFC, in accordance with single cell recordings in non-human primates, does indeed produce such a signal when an anticipated reward does not occur, provided the omission of reward is relevant for subsequent behavior.
